# Safety of tenecteplase vs. alteplase in telestroke: a large multistate experience (STAT)

**DOI:** 10.3389/fneur.2024.1514915

**Published:** 2025-01-08

**Authors:** Morgan Figurelle, Sandro Corti, Oleg Collins, Lan Gao, Amanda Avila, Kristie Delfino, Laurie Mayer, Theresa Sevilis

**Affiliations:** ^1^TeleSpecialists, LLC, Fort Myers, FL, United States; ^2^Department of Mathematics, University of Tennessee at Chattanooga, Chattanooga, TN, United States

**Keywords:** tenecteplase, alteplase, telestroke, acute stroke care, thrombolytics

## Abstract

**Introduction:**

Prompt treatment with IV thrombolytics (IVT) in acute ischemic stroke (AIS) patients is critical for improved recovery and survival. Recently, hospital systems have switched to the IVT tenecteplase (TNK) instead of the FDA-approved alteplase (tPA) for treatment. Multiple studies and meta-analyses evaluating the efficacy and safety of TNK demonstrate similar or superior outcomes when compared to tPA. TNK is not FDA-approved for treatment, which has led to hesitation in its use and increased attention on its complication profile, including the risk of intracranial hemorrhage (ICH).

**Methods:**

Data from AIS consults conducted in the emergency departments of 220 facilities across 26 states, between 1 January 2022 and 31 May 2023, were extracted from the TeleCare by TeleSpecialists™ database. The encounters were reviewed for IVT candidates, door-to-needle (DTN) time, type of IVT administered, use of advanced imaging, presence of LVO, occurrence and type of complications, complication type, symptomatic ICH, and the ECASS II ICH score.

**Results:**

A total of 2,305 TNK patients and 3,337 tPA patients were extracted. DTN times were faster (37 min vs. 42 min, *p* < 0.0001), and more total complications were observed in the TNK group (87 vs. 80, *p* = 0.0035). In non-LVO IVT patients, the TNK group had more complications (57 vs. 47, *p* = 0.0078), specifically ICH (48 vs. 35, *p* = 0.0036). No statistically significant difference in the incidence of ICH was observed between the TNK group and the tPA group (21 vs. 18, *p* = 0.07). In IVT patients not accepted for NIR, the TNK group had more complications (77 vs. 69, *p* = 0.005), specifically ICH (63 vs. 51, *p* = 0.0026). In IVT patients accepted for NIR, no significant differences were observed. There were no statistically significant differences in symptomatic ICH between the groups.

**Conclusion:**

The TNK group was found to have significantly more complications, including ICH, than the tPA group driven by non-LVO patients. A closer analysis of the potential for increased risk to non-LVO patients is warranted based on this large, multistate, and multi-hospital system study.

## Introduction

Stroke is one of the leading causes of death and disability in the United States and globally. Prompt recognition of symptoms and treatment with IV thrombolytics in acute ischemic stroke patients is critical for improved recovery and survival (1). Until recently, the gold standard of thrombolytic treatment was alteplase (tPA), which was approved by the FDA in 1996 and remains the only FDA-approved treatment for acute stroke patients in the United States. However, both small community hospitals and large tertiary care systems have been transitioning to the newer modified form of tPA, tenecteplase (TNK), for thrombolytic treatment due to its cost-effectiveness and ease of administration. TNK has a longer half-life than tPA; therefore, it can be administered in a single bolus within seconds rather than needing an hour-long continuous infusion. It also appears to have an increased specificity to fibrin within clots, theoretically leading to more effective thrombus lysis (2).

Multiple studies and meta-analyses evaluating the efficacy and safety of TNK demonstrate similar or superior outcomes when compared to tPA (3). The most recent acute stroke guidelines from the American Heart Association have also incorporated the use of tenecteplase in the setting where a mechanical thrombectomy is planned (4). Despite these studies, the lack of FDA approval for TNK has led to hesitation in its use and increased attention concerning its complication profile, particularly intracranial hemorrhage (ICH).

The purpose of this study was to compare TNK and tPA complications in the community setting to add to the data in randomized controlled trials, specifically to see whether stroke types such as large vessel occlusions (LVOs) or supplemental invasive treatment with neurointerventional radiology (NIR) affect bleeding rates.

## Methods

Data from acute stroke consultations conducted in the emergency departments of 220 facilities across 26 states, between 1 January 2022 and 31 May 2023, were extracted from the TeleCare by TeleSpecialists™ database. This database comprises prospectively collected data that were retrospectively reviewed. The criteria for treatment with thrombolytic was limited to the 4.5-hour from last known normal time window for all the patients included in the study. The encounters were reviewed for age, gender, National Institutes of Health Stroke Scale (NIHSS) score, premorbid modified Rankin Score (pre-mRS), race, ethnicity, IV thrombolytic candidacy, door-to-needle (DTN) time, type of thrombolytics used, advanced imaging, presence of LVO, complication occurrence, type of complications, presence of ICH, symptomatic ICH, and the ECASS II ICH score. Once a hemorrhagic complication was identified, stroke neurologists reviewed images in PACS to validate the ECASS II ICH Score as part of the standard quality review process per the TeleSpecialists, LLC quality improvement project following the transition to TNK.

### Statistical analysis

For the statistical analysis, this study compared demographics and clinical outcome variables between the two groups of simultaneously collected stroke patients who received either tPA or TNK as the sole thrombolytic agent, per facility protocol. A total of 5,642 patients were included (TNK: 2,305 and tPA: 3,337). Continuous variables (e.g., age and DTN times) were compared using Student’s *t*-test for normally distributed data and the Mann–Whitney *U*-test for non-normally distributed data. Categorical variables (e.g., complications and symptomatic intracranial hemorrhage [ICH]) were analyzed using Pearson’s chi-square test. Subgroup analyses were conducted based on the presence of large vessel occlusion (LVO) and candidacy for neurointervention (NIR). Patients were further categorized by LVO status within each group, allowing for comparisons of both continuous and categorical variables. Statistical significance was defined as a *p*-value <0.05. All analyses were performed using R version 4.3.1.

## Results

**Figure 1:**
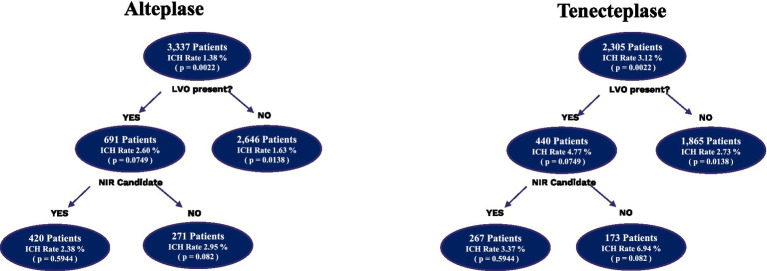
Subgroup analysis of intracranial hemorrhages in alteplase vs. tenecteplase patients. Depiction of the number of patients who received alteplase and tenecteplase subdivided by the presence of LVO and the intracranial hemorrhage rates. The LVO patients were then further subdivided into whether they were accepted for neurointervention or not and the associated intracranial hemorrhage rates. ICH, intracranial hemorrhage.

A total of 5,642 patients were included, with 2,305 in the TNK group and 3,337 in the tPA group (Figure 1). Within patient demographics, there was a statistically significant difference in age, with the TNK group being slightly older (Table 1). DTN times were significantly faster in the TNK group. However, significantly more total complications and more ICH were observed. There was no statistically significant difference in the rate of symptomatic ICH, which is consistent with previous smaller studies.

**Table 1:** Comparison of alteplase vs. tenecteplase groups.

	Alteplase (*n* = 3,337)	Tenecteplase (*n* = 2,305)	*p*-value
Baseline characteristics			
Age, mean (SD)	65.63 ± 15.71	66.70 ± 15.76	**0.012**
Gender: female n (%)	1,681 (50.39%)	1,183 (51.35%)	0.4973
NIHSS median (IQR)	6 (3.00, 10.00)	6 (3.00, 11.00)	0.7781
Pre-mRS median (IQR)	0.00 (0.00, 0.00)	0.00 (0.00, 0.00)	**0.0285**
Race			
Asian *n* (%)	25 (1.70%)	22 (1.45%)	0.2056
Black *n* (%)	269 (18.32%)	235 (15.53%)	
Caucasian *n* (%)	1,173 (79.90%)	1,255 (82.95%)	
Hawaiian or other PI *n* (%)	1 (0.07%)	1 (0.07%)	
Ethnicity			
Hispanic *n* (%)	209 (27.46%)	77 (10.32%)	**<0.0001**
Non-hispanic *n* (%)	552 (72.54%)	669 (89.68%)	
Total complications			
Yes *n* (%)	80 (2.40%)	87 (3.77%)	**0.0035**
No *n* (%)	3,257 (97.60%)	2,218 (96.23%)	
Individual complications			
Angioedema *n* (%)	4 (0.12%)	3 (0.13%)	0.9999
GIB *n* (%)	2 (0.06%)	0 (0.00%)	0.6482
ICH *n* (%)	61 (1.83%)	72 (3.12%)	**0.0022**
Oral bleeding *n* (%)	0 (0.00%)	1 (0.04%)	0.8524
Other *n* (%)	13 (0.39%)	11 (0.48%)	0.7724
Symptomatic ICH rate			
Yes *n* (%)	26 (42.62%)	32 (44.44%)	0.9716
No *n* (%)	35 (57.38%)	40 (55.56%)	
Type of hemorrhage			
HI1 *n* (%)	9 (14.75%)	10 (13.89%)	0.9999
HI2 *n* (%)	7 (11.48%)	10 (13.89%)	0.877
PH1 *n* (%)	11 (18.03%)	12 (16.67%)	0.9999
PH2 *n* (%)	28 (45.90%)	33 (45.83%)	0.9999
SAH *n* (%)	5 (8.20%)	7 (9.72%)	0.9982
SDH *n* (%)	1 (1.64%)	0 (0.00%)	0.9336
DTN times			
Median (IQR)	41.82 (31.15, 56.55)	37.00 (26.39, 51.00)	**<0.0001**

Data presented as *n* (%) except for median DTN, median arrival to notification, last known normal to arrival, median NIHSS, median p-mRS [median (IQR)], and age [mean (SD)]. Bold values indicate a significance at *p* < 0.05. tPA, alteplase; TNK, tenecteplase; DTN, door to needle; min, minutes; NIHSS, National Institute of Health Stroke Scale; p-mRS, Premorbid Modified Rankin Scale; ICH, intracranial hemorrhagel; GIB, gastrointestinal bleeding; SAH, subarachnoid hemorrhage; SDH, subdural hematoma; HI, hemorrhagic infarction; PH, parenchymal hematoma.

A total of 4,511 patients did not have an LVO present, with 1,865 in the TNK group and 2,646 in the tPA group. The baseline NIHSS score in both groups was 5. There were some statistical differences within the demographics of this cohort (Table 2), with older age in the TNK group and significantly more Hispanic patients in the tPA group. The DTN time was significantly faster in the TNK group. There were more total complications, specifically ICH, but no statistical difference in symptomatic ICH.

**Table 2:** Comparison of variables between alteplase and tenecteplase subgroups of non-LVO patients.

	Alteplase (*n* = 2,646)	Tenecteplase (*n* = 1,865)	*p*-value
Baseline characteristics			
Age, mean (SD)	64.52 ± 15.77	65.81 ± 15.87	**0.0071**
Gender: female n (%)	1,376 (52.02%)	966 (51.82%)	0.9194
NIHSS median (IQR)	5.00 (3.00, 8.00)	5 (3.00, 8.00)	0.8903
Pre-mRS median (IQR)	0 (0.00, 0.00)	0.00 (0.00, 0.00)	0.2729
Race			
Asian *n* (%)	16 (1.36%)	13 (1.07%)	0.1997
Black *n* (%)	213 (18.10%)	186 (15.37%)	
Caucasian *n* (%)	948 (80.54%)	1,010 (83.47%)	
Hawaiian or Other PI *n* (%)	0 (0.00%)	1 (0.08%)	
Ethnicity			
Hispanic *n* (%)	166 (26.69%)	60 (10.15%)	**<0.0001**
Non-hispanic *n* (%)	456 (73.31%)	531 (89.85%)	
Total complications			
Yes *n* (%)	57 (2.15%)	63 (3.38%)	**0.0155**
No *n* (%)	2,589 (97.85%)	1,802 (96.62%)	
Individual complications			
Angioedema *n* (%)	3 (0.11%)	3 (0.16%)	0.9872
GIB *n* (%)	1 (0.04%)	0 (0.00%)	0.9999
ICH *n* (%)	43 (1.63%)	51 (2.73%)	**0.0138**
Oral bleeding *n* (%)	0 (0.00%)	0 (0.00%)	
Other *n* (%)	10 (0.38%)	9 (0.48%)	0.7634
Symptomatic ICH rate			
Yes *n* (%)	21 (48.84%)	25 (49.02%)	0.9999
No *n* (%)	22 (51.16%)	26 (50.98%)	
Type of hemorrhage			
HI1 *n* (%)	4 (9.30%)	6 (11.76%)	0.9601
HI2 *n* (%)	4 (9.30%)	6 (11.76%)	0.9601
PH1 *n* (%)	9 (20.93%)	8 (15.69%)	0.6972
PH2 *n* (%)	21 (48.84%)	26 (50.98%)	0.9999
SAH *n* (%)	4 (9.30%)	5 (9.80%)	0.9999
SDH *n* (%)	1 (2.33%)	0 (0.00%)	0.9316
DTN times			
Median (IQR)	43.00 (32.32, 57.98)	37.08 (27.00, 50.97)	**<0.0001**

Data presented as *n* (%) except for median DTN, median arrival to notification, last known normal to arrival, median NIHSS, median p-mRS [median (IQR)], and age [mean (SD)]. Bold values indicate a significance at p < 0.05. Abbreviations: tPA: alteplase, TNK: tenecteplase, DTN: door to needle, min: minutes, NIHSS: National Institute of Health Stroke Scale, p-mRS: Premorbid Modified Rankin Scale, ICH: intracranial hemorrhage, GIB: gastrointestinal bleeding, SAH: subarachnoid hemorrhage, SDH: subdural hematoma, HI: hemorrhagic infarction, PH: parenchymal hematoma.

There were 1,131 patients identified with an LVO. Of these patients, 691 patients received tPA and 440 patients received TNK. The demographics showed (Table 3) that the tPA group had significantly more Hispanic patients. The baseline NIHSS score in both groups was 13. There were no significant differences in complications, ICH, or in DTN time. Of the 1,131 patients with an LVO, 687 patients were accepted for neurointervention, while 444 patients were not. A total of 420 NIR patients were administered tPA and 267 patients were administered TNK, and no significant differences in demographics, complications, or DTN time were observed (Table 4). The baseline NIHSS score in both groups was 14.

**Table 3:** Comparison of variables between alteplase and tenecteplase subgroups with an LVO.

	Alteplase (*n* = 691)	Tenecteplase (*n* = 440)	*p*-value
Baseline characteristics			
Age, mean (SD)	69.89 ± 14.72	70.50 ± 14.67	0.4976
Gender: female *n* (%)	305 (44.14%)	217 (49.32%)	0.1005
NIHSS median (IQR)	13 (7.00, 19.00)	13.00 (8.00, 19.25)	0.3568
Pre-mRS median (IQR)	0 (0.00, 0.00)	0.00 (0.00, 1.00)	**0.0063**
Race			
Asian *n* (%)	9 (3.09%)	9 (2.97%)	0.5568
Black *n* (%)	56 (19.24%)	49 (16.17%)	
Caucasian *n* (%)	225 (77.32%)	245 (80.86%)	
Hawaiian or other PI *n* (%)	1 (0.34%)	0 (0.00%)	
Ethnicity			
Hispanic *n* (%)	43 (30.94%)	17 (10.97%)	**<0.0001**
Non-hispanic *n* (%)	96 (69.06%)	138 (89.03%)	
Total complications			
Yes *n* (%)	23 (3.33%)	24 (5.45%)	0.111
No *n* (%)	668 (96.67%)	416 (94.55%)	
Individual complications			
Angioedema *n* (%)	1 (0.14%)	0 (0.00%)	0.9999
GIB *n* (%)	1 (0.14%)	0 (0.00%)	0.9999
ICH *n* (%)	18 (2.60%)	21 (4.77%)	0.0749
Oral bleeding *n* (%)	0 (0.00%)	1 (0.23%)	0.8199
Other *n* (%)	3 (0.43%)	2 (0.45%)	0.9999
Symptomatic ICH rate			
Yes *n* (%)	5 (27.78%)	7 (33.33%)	0.9786
No *n* (%)	13 (72.22%)	14 (66.67%)	
Type of hemorrhage			
HI1 *n* (%)	5 (27.78%)	4 (19.05%)	0.7919
HI2 *n* (%)	3 (16.67%)	4 (19.05%)	0.9999
PH1 *n* (%)	2 (11.11%)	4 (19.05%)	0.8106
PH2 *n* (%)	7 (38.89%)	7 (33.33%)	0.9795
SAH *n* (%)	1 (5.56%)	2 (9.52%)	0.9999
SDH *n* (%)	0 (0.00%)	0 (0.00%)	0.631
DTN times			
Median (IQR)	37.10 (27.38, 51.22)	35.48 (24.96, 51.80)	0.1262

Data presented as n (%) except for median DTN, median arrival to notification, last known normal to arrival, median NIHSS, median p-mRS [median (IQR)], and age [mean (SD)]. Bold values indicate a significance at *p* < 0.05. tPA, alteplase; TNK, tenecteplase; DTN, door to needle; min, minutes; NIHSS, National Institute of Health Stroke Scale; p-mRS, Premorbid Modified Rankin Scale; ICH, intracranial hemorrhage; GIB, gastrointestinal bleeding; SAH, subarachnoid hemorrhage; SDH, subdural hematoma; HI, hemorrhagic infarction; PH, parenchymal hematoma.

**Table 4:** Comparison of variables between alteplase and tenecteplase subgroups with LVO who were accepted for neurointervention.

	Alteplase (*n* = 420)	Tenecteplase (*n* = 267)	*p*-value
Baseline characteristics			
Age mean (SD)	69.50 ± 15.06	68.97 ± 14.40	0.6437
Gender: female *n* (%)	185 (44.05%)	128 (47.94%)	0.3576
NIHSS median (IQR)	14.00 (9.00, 19.00)	14 (10.00, 20.00)	0.3632
Pre-mRS median (IQR)	0.00 (0.00, 0.00)	0 (0.00, 1.00)	0.0884
Race			
Asian *n* (%)	6 (3.05%)	3 (1.69%)	0.5705
Black *n* (%)	39 (19.80%)	31 (17.51%)	
Caucasian *n* (%)	152 (77.16%)	143 (80.79%)	
Hawaiian or other PI *n* (%)	0 (0.00%)	0 (0.00%)	
Ethnicity			
Hispanic *n* (%)	24 (25.00%)	13 (13.83%)	0.0783
Non-hispanic *n* (%)	72 (75.00%)	81 (86.17%)	
Total complications			
Yes *n* (%)	11 (2.62%)	10 (3.75%)	0.7033
No *n* (%)	409 (97.38%)	257 (96.25%)	
Individual complications			
Angioedema *n* (%)	0 (0.00%)	0 (0.00%)	
GIB *n* (%)	0 (0.00%)	0 (0.00%)	
ICH *n* (%)	10 (2.38%)	9 (3.37%)	0.5944
Oral bleeding *n* (%)	0 (0.00%)	0 (0.00%)	
Other *n* (%)	1 (0.24%)	1 (0.37%)	0.9999
Symptomatic ICH rate			
Yes *n* (%)	2 (20.00%)	3 (33.33%)	0.8908
No *n* (%)	8 (80.00%)	6 (66.67%)	
Type of hemorrhage			
HI1 *n* (%)	2 (20.00%)	3 (33.33%)	0.8908
HI2 *n* (%)	2 (20.00%)	1 (11.11%)	0.9999
PH1 *n* (%)	1 (10.00%)	1 (11.11%)	0.9999
PH2 *n* (%)	4 (40.00%)	2 (22.22%)	0.7352
SAH *n* (%)	1 (10.00%)	2 (22.22%)	0.9208
SDH *n* (%)	0 (0.00%)	0 (0.00%)	0.8185
DTN times			
Median (IQR)	36.82 (26.92, 49.08)	34.00 (24.00, 47.96)	0.1046

Data presented as *n* (%) except for median DTN, median arrival to notification, last known normal to arrival, median NIHSS, median p-mRS [median (IQR)], and age [mean (SD)]. Bold values indicate a significance at *p* < 0.05. tPA, alteplase; TNK, tenecteplase; DTN, door to needle; min, minutes; NIHSS, National Institute of Health Stroke Scale; p-mRS, Premorbid Modified Rankin Scale; ICH, intracranial hemorrhage; GIB, gastrointestinal bleeding; SAH, subarachnoid hemorrhage; SDH, subdural hematoma; HI, hemorrhagic infarction; PH, parenchymal hematoma.

In the 444 patients who had an LVO and were not candidates for NIR, 271 patients were administered tPA and 173 patients were administered TNK. TNK patients were older than tPA patients, and tPA patients were significantly more likely to be Hispanic (Table 5). The baseline NIHSS score in both groups was 10. There were no significant differences in complications, ICH, or in DTN time. There were no statistically significant differences in symptomatic ICH between all groups.

**Table 5:** Comparison of variables between alteplase and tenecteplase subgroups with LVO and no neurointervention.

	**Alteplase** (*N* = 271)	**Tenecteplase** (*N* = 173)	***p*-value**
Baseline characteristics			
Age mean (SD)	70.50 ± 14.18	72.86 ± 14.81	0.0964
Gender: female n (%)	120 (44.28%)	89 (51.45%)	0.1684
NIHSS median (IQR)	10 (5.00, 17.00)	10 (5.00, 18.00)	0.8104
Pre-mRS median (IQR)	0 (0.00, 1.00)	0 (0.00, 2.00)	**0.0289**
Race			
Asian *n* (%)	3 (3.19%)	6 (4.76%)	0.5267
Black *n* (%)	17 (18.09%)	18 (14.29%)	
Caucasian *n* (%)	73 (77.66%)	102 (80.95%)	
Hawaiian or other PI *n* (%)	1 (1.06%)	0 (0.00%)	
Ethnicity			
Hispanic *n* (%)	19 (44.19%)	4 (6.56%)	**<0.0001**
Non-hispanic *n* (%)	24 (55.81%)	57 (93.44%)	
Total complications			
Yes *n* (%)	12 (4.43%)	14 (8.09%)	0.1626
No *n* (%)	259 (95.57%)	159 (91.91%)	
Individual complications			
Angioedema *n* (%)	1 (0.37%)	0 (0.00%)	0.9999
GIB *n* (%)	1 (0.37%)	0 (0.00%)	0.9999
ICH *n* (%)	8 (2.95%)	12 (6.94%)	0.082
Oral bleeding *n* (%)	0 (0.00%)	1 (0.58%)	0.8208
Other *n* (%)	2 (0.74%)	1 (0.58%)	0.9999
Symptomatic ICH rate			
Yes *n* (%)	3 (37.50%)	4 (33.33%)	0.9999
No *n* (%)	5 (62.50%)	8 (66.67%)	
Type of hemorrhage			
HI1 *n* (%)	3 (37.50%)	1 (8.33%)	0.3044
HI2 *n* (%)	1 (12.50%)	3 (25.00%)	0.9092
PH1 *n* (%)	1 (12.50%)	3 (25.00%)	0.9092
PH2 *n* (%)	3 (37.50%)	5 (41.67%)	0.9999
SAH *n* (%)	0 (0.00%)	0 (0.00%)	0.9982
SDH *n* (%)	0 (0.00%)	0 (0.00%)	0.9336
DTN times			
Median (IQR)	37.48 (28.23, 55.04)	39.57 (26.17, 57.00)	0.7113

Data presented as *N* (%) except for median DTN, median arrival to notification, last known normal to arrival, median NIHSS, median p-mRS [median (IQR)], and age [mean (SD)]. Bold values indicate a significance at *p* < 0.05. tPA, alteplase; TNK, tenecteplase; DTN, door to needle; min, minutes; NIHSS, National Institute of Health Stroke Scale; p-mRS, Premorbid Modified Rankin Scale; ICH, intracranial hemorrhage; GIB, gastrointestinal bleeding; SAH, subarachnoid hemorrhage; SDH, subdural hematoma; HI, hemorrhagic infarction; PH, parenchymal hematoma.

## Conclusion

This study is the largest, non-randomized, community-based study of the complications of tPA vs. TNK in the acute stroke setting. Overall, DTN times were significantly faster in the TNK group, which is advantageous when considering the importance of expedited treatment to improve patient outcomes (5). With studies showing non-inferiority and the utilization of a non-FDA-approved treatment when an FDA-approved treatment is available, it is important to have detailed data on the complication profile. This large sample demonstrated an increased rate of total complications, though it is important to note that the complication rates within both groups remained low (tPA 2.40% and TNK 3.77%) compared to the most cited complication rate of 6.4% (6). The only significant difference observed was in the rate of intracranial hemorrhages (tPA 1.83% and TNK 3.12%), with no significant differences in the rates of angioedema, GI bleeding, oral bleeding, or other complications. One of the most important findings is that despite the overall ICH rate being higher with TNK, there was no significant difference in symptomatic ICHs.

Randomized controlled trials have shown non-inferior outcomes and a trend toward more intracranial hemorrhages but not symptomatic hemorrhages (3). This large real-world study shows that the increased hemorrhage rate is statistically significant with a larger sample size, but there is still no significant difference in symptomatic hemorrhage. While these data do not include outcomes, they do provide unique insight into the complications profile.

The ability to compare the LVO vs. non-LVO groups, as well as those who were accepted for neurointervention vs. those who were not, shows some interesting insights. Surprisingly, the difference in ICH was caused by the increased rate of ICH in non-LVO patients receiving TNK. These patients tend to have lower NIHSS scores and smaller stroke volumes. This may explain why there are fewer symptomatic ICHs in this patient population. However, there was no significant difference in the ECASS II score assigned to the hemorrhages, and no cases of subarachnoid hemorrhages or subdural hematomas were observed in this group. There was no significant difference in hemorrhage grading volumes among the subgroups or the primary analysis.

With regard to symptomatic ICH, there is no significant difference between TNK and tPA, which is an important finding with the TNK transition trend. Although there were no significant differences in symptomatic intracranial hemorrhage between the groups, the increased ICH rate in non-LVO patients with TNK stands out in this study as a potentially pertinent finding. Future research targeting the non-LVO population receiving thrombolytics in the 4.5-h time window is warranted, focusing on outcomes.

## Data Availability

The raw data supporting the conclusions of this article will be made available by the authors, without undue reservation.

## References

[1] Dhar N Kumar M Tiwari A Desai I Madhaw G Kumar N. Tenecteplase and Alteplase for thrombolysis of acute ischemic stroke within 4.5 hours: an efficacy and safety study. Ann Indian Acad Neurol. (2022) 25:897–901. doi: 10.4103/aian.aian_1127_21, PMID: 36561006 PMC9764915

[2] Singh N Almekhlafi M Bala F Ademola A Coutts S Deschaintre Y. Effect of time to thrombolysis on clinical outcomes in patients with acute ischemic stroke treated with Tenecteplase compared to Alteplase: analysis from the AcT randomized controlled trial. Stroke. (2023) 54:2766–75. doi: 10.1161/STROKEAHA.123.044267, PMID: 37800372

[3] Wang Y Li S Pan Y Li H Parsons MW Campbell BCV. TRACE-2 investigators. Tenecteplase versus alteplase in acute ischaemic cerebrovascular events (TRACE-2): a phase 3, multicentre, open-label, randomised controlled, non-inferiority trial. Lancet. (2023) 401:645–54. doi: 10.1016/S0140-6736(22)02600-9, PMID: 36774935

[4] Powers WJ Rabinstein AA Ackerson T Adeoye OM Bambakidis NC Becker K. Guidelines for the early Management of Patients with Acute Ischemic Stroke: 2019 update to the 2018 guidelines for the early Management of Acute Ischemic Stroke: a guideline for healthcare professionals from the American Heart Association/American Stroke Association. Stroke. (2019) 50:e344–418. doi: 10.1161/STR.0000000000000211 31662037

[5] Saver JL Fonarow GC Smith EE Reeves MJ Grau-Sepulveda MV Pan W. Time to treatment with intravenous tissue plasminogen activator and outcome from acute ischemic stroke. JAMA. (2013) 309:2480–8. doi: 10.1001/jama.2013.6959, PMID: 23780461

[6] National Institute of Neurological Disorders and Stroke rt-PA stroke study group. Tissue plasminogen activator for acute ischemic stroke. N Engl J Med. (1995) 333:1581–8. doi: 10.1056/NEJM199512143332401, PMID: 7477192

